# Cerebral Hemodynamic Evaluation of Main Cerebral Vessels in Epileptic Patients Based on Transcranial Doppler

**DOI:** 10.3389/fneur.2021.639472

**Published:** 2021-05-20

**Authors:** Jihong Meng, Chun Li, Weining Ma

**Affiliations:** ^1^Department of Neurosurgery, Shengjing Hospital of China Medical University, Shenyang, China; ^2^Department of Pediatrics, Shengjing Hospital of China Medical University, Shenyang, China

**Keywords:** peak flow velocity, transcranial doppler, epilepsy, FDG 18-PET, interictal electroencephalogram

## Abstract

**Objective:** To study whether there is a difference in peak and mean blood flow velocity between the left and right major cerebral vessels in patients with epilepsy.

**Methods:** Sixteen patients with epilepsy underwent FDG^18^-PET-CT (PET) scan and electroencephalogram (EEG) examinations. Transcranial Doppler (TCD) was used to detect the peak flow velocity (PFV), mean flow velocity (MFV), and other hemodynamic indicators of bilateral anterior, middle, and posterior cerebral arteries in each patient. According to different patterns of the PET or interictal EEG, the differences in PFV, and MFV of corresponding vessels on both sides under different patterns were compared.

**Results:** According to the PET of the low-metabolism region corresponding to the supplying artery, the PFV and MFV of the supplying artery in the low-metabolism region were lower than the value of the corresponding contralateral vessel. The PFV and MFV on the low metabolic side of PET were lower than that of the corresponding vessels on the opposite side. The PFV and MFV on the discharge side of interictal EEG were also lower than the PFV and MFV of the corresponding vessels on the opposite side. The MFV of posterior cerebral artery on the low metabolic side of PET or the interictal discharge side was significantly different from that of the contralateral vessels (*P* < *0.05*). However, the other aforementioned differences in PFV and MFV did not achieve statistical significance.

**Conclusion:** In epileptic patients, the PFV and MFV of main cerebral vessels on the PET hypometabolized side or the interictal discharge side was lower than that of corresponding vessels on the contralateral side. To some extent, the difference in the MFV of PCA between the bilateral sides can facilitate the lateral diagnosis of the epileptogenic zone.

## Introduction

Epilepsy is a common neurological disease, positron emission tomography (FDG^18^-PET-CT) and electroencephalogram (EEG) are the conventional means of locating and diagnosing epilepsy. It is currently believed that the low metabolism region (1) in non-seizure phase FDG^18^-PET-CT (PET) examination and the discharge active region in interictal EEG are consistent with the range and laterality of the epileptogenic zone. In previous studies on cerebral blood flow (CBF) in epilepsy using PET, it was found that compared with the normal control group, CBF in the multi-brain areas of the lesion side was significantly decreased (2) and CBF in the interictal discharge areas was increased (3) in patients with temporal lobe epilepsy. In addition, the study (4) showed that the epileptic patient's dynamic cerebral autoregulation (dCA) declines, especially in patients with a slow wave in interictal EEG. All of these studies demonstrated that the cerebral hemodynamics of epileptic patients were abnormal compared with that of non-epilectics. To our knowledge, there have been few studies comparing differences in cerebral hemodynamics between the main vessels of both sides in the same epileptic patient. Transcranial Doppler (TCD), a technique that uses ultrasound Doppler effect to detect the hemodynamics of the main arteries in the brain and skull base, evaluates the flow using velocity. In this study, we used a non-invasive TCD method to detect the brain's hemodynamic values, including the peak flow velocity (PFV) and mean flow velocity (MFV) of the anterior (ACA), middle (MCA), and posterior (PCA) cerebral arteries on both sides of the epileptic patient. The data were analyzed according to the metabolism of FDG^18−^PET-CT examination, discharge pattern of interictal EEG, and EEG background. In addition, differences in PFV and MFV were compared between the abnormal and contralateral sides of PET metabolism and interictal EEG discharge. Differences in the PFV and MFV may provide additional assistance for the localization and lateral diagnosis of epilepsy.

## Materials and Methods

### Subjects

Sixteen subjects with epilepsy from the Neurosurgery Department of Shengjing Hospital of China Medical University were enrolled in this study. All enrolled cases were consistent with the diagnosis of epilepsy in terms of EEG and/or symptoms, and all had vascular examinations, such as magnetic resonance angiography (MRA), hippocampal magnetic resonance imaging (MRI), or thin-layer enhanced MRI. TCD was used to obtain the PFV and MFV. All patients underwent PET and EEG examinations. Approval for this study was obtained from the Ethics Committee of Shengjing Hospital of China Medical University and informed consent was obtained from all patients.

### Transcranial Doppler (TCD) Detection Method

Hemodynamic indices of the bilateral ACA, MCA, and PCA were measured in all patients using the digital dual channel, multi-depth transcranial Doppler instrument and pulse Doppler probe with a frequency of 1.6 Hz (MVU6203, Delica, China). TCD testing was performed by the same trained and experienced physician. In the supine temporal window, the bilateral ACA, MCA, and PCA were examined and the PFV, end-diastolic flow velocity, and peripheral vascular resistance index (PI) were recorded. MFV were calculated by PFV and end-diastolic flow velocity. The probe angles of the ACA and MCA were slightly overhead, with a probe Angle of 30–45 degrees, while the probe angles of PCA were slightly lower. The artery depth was 60–70 mm for the ACA, 40–65 mm for the MCA, and 60–70 mm for the PCA. None of the patients had vascular stenosis.

### The PET Hypometabolized Areas Correspond to the Supplying Arteries

The [^18^F] FDG PET CT scan was acquired within interictal period for every patient. The low metabolic region of PET was determined by observing the symmetry of the metabolic level on both sides of the hemispheres visually. Low metabolism of the frontal pole or medial frontal lobe corresponds to the ACA; low metabolism of the frontal lobe, temporal lobe, or parietal lobe cortex around the lateral fissure corresponds to the MCA; and low metabolism of hippocampus or occipital lobe corresponds to the PCA.

### Interictal EEG Patterns

Scalp EEG (64 channels, Nicolet, USA) with a 10–20 system was used for monitoring. At least 24 h of interictal EEG should be recorded, which must include both waking and sleeping periods. EEG discharge patterns were: unilateral discharge (discharge is located in one hemisphere); bilateral discharge (synchronous and asynchronous discharge of both hemispheres); and no discharge. An occipital rhythm < 9 Hz was defined as the background rhythm slowing down.

### Statistical Analysis

Data analysis was performed using SPSS v.17.0 (IBM, Chicago, IL). Categorical data are presented as the average and an independent sample *t*-test was used to compare continuous data between two groups. A *P* < 0.05 was considered a statistically significant difference.

## Results

### Characteristics of Subjects

**Figure 1:**
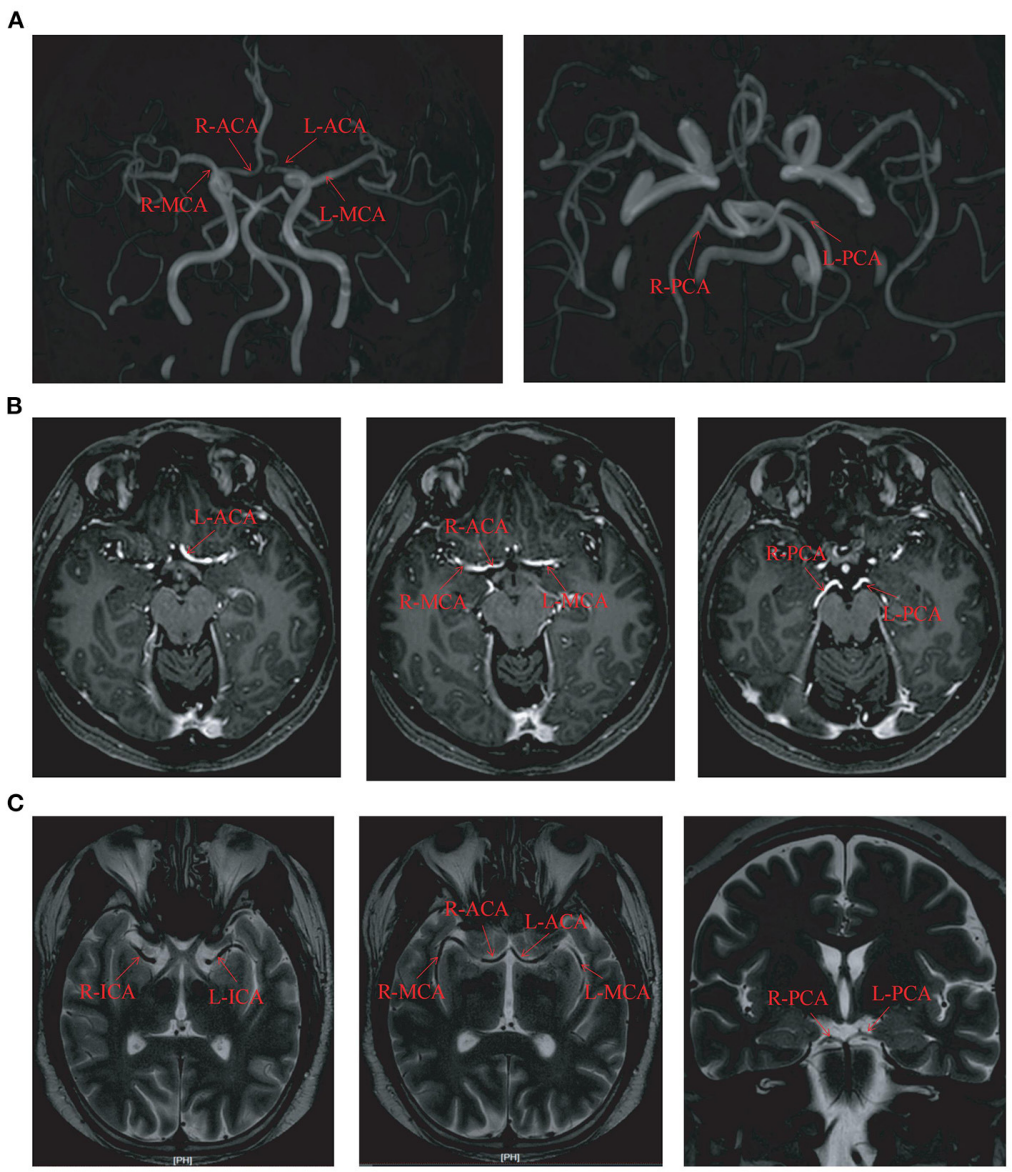
The circle of Willis imaging in some cases. **(A)** The magnetic resonance angiography imaging in case number 3. **(B)** The hippocampal magnetic resonance imaging in case number 2. **(C)** The thin-layer enhanced MRI imaging in case number 15. L, left; R, right; ACA, anterior cerebral artery; MCA, middle cerebral artery; PCA, posterior cerebral artery; ICA, Internal carotid artery.

Sixteen patients (9 males and 7 females) with an average age of 33 years (range, 3–64 years) were enrolled in this study. Their average duration of epilepsy was 13 years (range, 1–38 years). Among the 16 patients with epilepsy, there were 7 cases of focal epilepsy, 7 cases of generalized epilepsy and 2 cases of combined generalized and focal epilepsy (Table 1). In addition, among the enrolled patients, the right MCA was narrower than the left in one patient, the right embryonic PCA in one patient, and the ACA, MCA, and PCA in the other cases were bilateral symmetrical (Table 1, Figure 1). PET examination showed that 14 patients had low metabolism in one side, one patient had low metabolism in multiple brain areas, and one patient with bilateral symmetrical metabolism (Table 2). In the interictal EEG, the discharge was confined to one side in five patients, there was bilateral discharge in 10 patients, and no discharge in one patient. The background rhythm slowed in three patients as shown in Table 2.

**Table 1:** The demographic and intracranial vascular anatomies of the cases.

**Case number**	**Age (years)**	**Sex**	**Duration (years)**	**Epilepsy types**	**Anti-epileptic drugs**	**L-ACA**	**L-MCA**	**L-PCA**	**R-ACA**	**R-MCA**	**R-PCA**	**AcoA**
1	28	Female	2	Combined Generalized and Focal	Levetiracetam	√	√	√	√	√	√	Open
2	32	Female	17	Generalized	Phenobarbital	√	√	√	√	√	√	Open
3	32	Female	28	Generalized	Phenytoin; Phenobarbital	√	√	√	√	√	√	Open
4	26	Male	2	Focal	Oxcarbazepine	√	√	√	√	√	√	Open
5	47	Female	1	Focal	–	√	√	√	√	√	√	–
6	3	Female	2	Combined Generalized and Focal	Levetiracetam; Sodium valproate; Topiramate	√	√	√	√	Narrow to the left	√	–
7	25	Male	3	Focal	Levetiracetam	√	√	√	√	√	Embryonic	–
8	60	Male	20	Focal	Phenytoin; Phenobarbital; Carbamazepine	√	√	√	√	√	√	Open
9	39	Male	33	Generalized	Phenobarbital; Carbamazepine; Oxcarbazepine; Lamotrigine	√	√	√	√	√	√	Open
10	64	Female	6	Generalized	Sodium valproate	√	√	√	√	√	√	Open
11	23	Female	5	Focal	–	√	√	√	√	√	√	Open
12	20	Male	10	Generalized	Topiramate; Levetiracetam	√	√	√	√	√	√	–
13	38	Male	38	Generalized	Phenobarbital	√	√	√	√	√	√	Open
14	26	Male	2	Focal	–	√	√	√	√	√	√	–
15	36	Male	24	Focal	Carbamazepine	√	√	√	√	√	√	–
16	26	Male	22	Generalized	Sodium valproate; Carbamazepine	√	√	√	√	√	√	–

*“√”, vessels were visible with bilateral symmetry; AcoA, anterior communicating artery; The opening of the AcoA was detected by TCD*.

**Table 2:** The FDG^18^-PET-CT and interictal discharge manifestations of the cases.

**Case number**	**Hypometabolic brain regions**	**The corresponding supplying arteries**	**EEG background (Hz)**	**Interictal discharge positions**	**Range**
1	Multi-regions	ACA, MCA, PCA	9.0–10.0	F7, F8, M1, M2	Bilateral
2	R-Temporal lobe cortex	R-MCA	10.0–11.0	M2	Unilateral
3	R-Frontal, temporal, and parietal lobe cortex	R-MCA	9.0–10.0	M1, M2	Bilateral
4	R-Temporal lobe and hippocampus cortex	R-MCA, R-PCA	10.0–11.0	F8, T4, M2	Unilateral
5	L-Temporal lobe cortex	L-MCA	9.0–10.0	M1, M2	Bilateral
6	R-Cerebral hemisphere	R-ACA, R-MCA, R-PCA	6.0–7.0	Fp2, F4	Unilateral
7	L-Occipital lobe	L-PCA	9.0	O1, T5	Unilateral
8	R-Temporal lobe cortex	R-MCA	9.0–10.0	M1, M2	Bilateral
9	R-Parietal lobe cortex	R-MCA	6.0–7.0	Multifocal discharges	Bilateral
10	R-Frontal and temporal lobe cortex	R-MCA	10–11.0	M1, M2	Bilateral
11	L-Temporal lobe and hippocampus cortex	L-MCA, L-PCA	9.0–10.0	Fp1, F7, M1	Unilateral
12	R-Frontal and temporal lobe cortex	R-MCA	7.0	Multifocal discharges	Bilateral
13	L-Temporal lobe cortex	L-MCA	9.0–10.0	F7, F8, M1, M2	Bilateral
14	Bilateral symmetry		9.0–10.0	Normal	Normal
15	R-Temporal lobe cortex	R-MCA	10.0–11.0	F7, F8, T4, T5, M1, M2, Fp2, F4, Fz	Bilateral
16	R-Temporal and occipital lobes	R-MCA, R-PCA	6.0–7.0	M1, M2	Bilateral

*L, left; R, right; ACA, anterior cerebral artery; MCA, middle cerebral artery; PCA, posterior cerebral artery; Electroencephalography (EEG) is performed using a 10–20 system*.

### Low Metabolism of PET and Flow Velocity of Bilateral Corresponding Arteries

**Figure 2:**
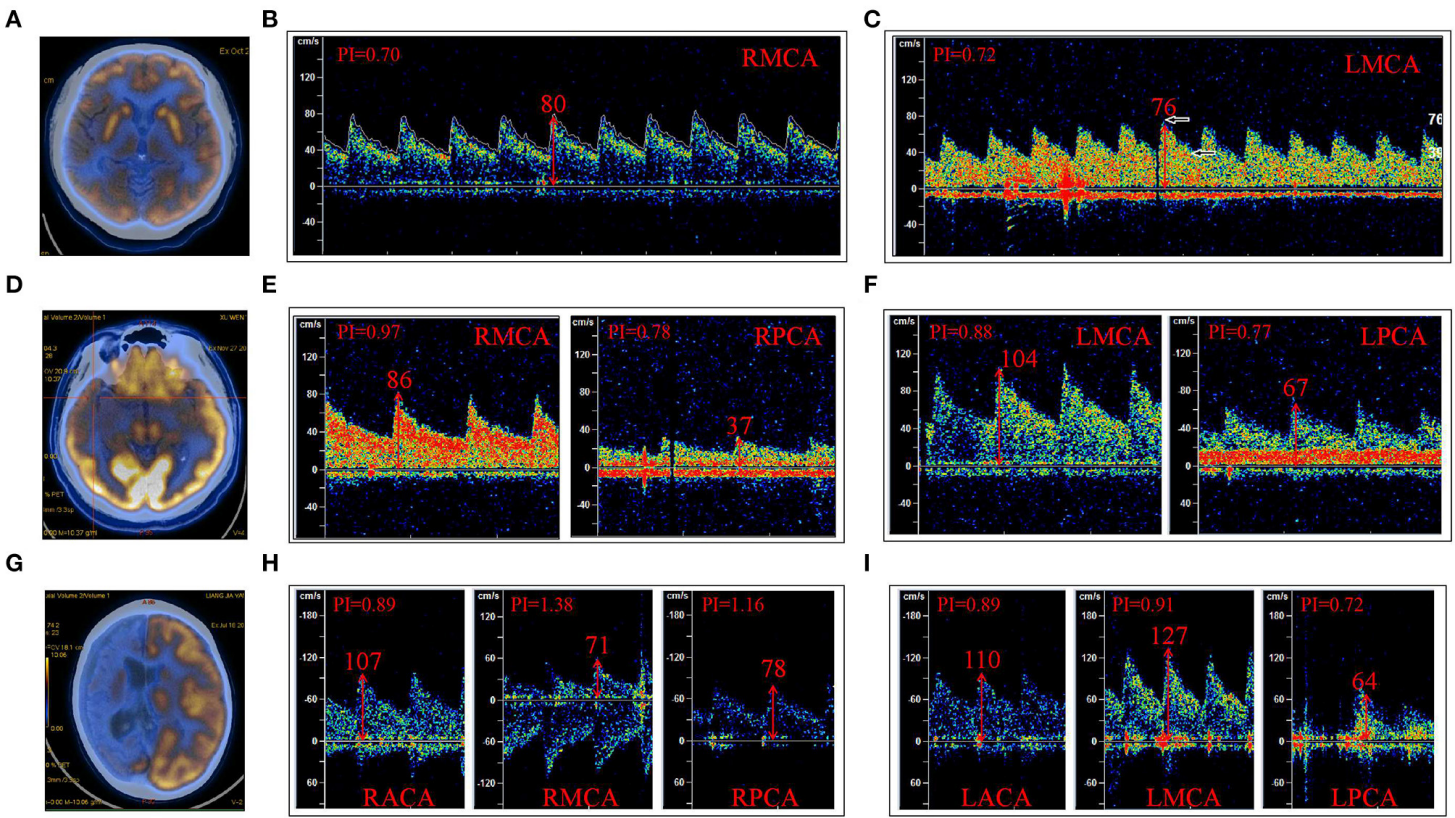
The low metabolic area of FDG^18^-PET-CT (PET) was divided according to the region of the supplying artery. **(A)** PET indicated a low metabolism in the right frontal and temporal cortex. **(B)** The PFV of RMCA was 80 cm/s; **(C)** The PFV of LMCA was 76 cm/s. **(D)** PET indicated low metabolism in the right temporal lobe and hippocampus; **(E)** The PFV of RMCA was 86 cm/s, and that of RPCA was 37 cm/s. **(F)** The PFV of LMCA was 104 cm/s and that of LPCA was 67 cm/s. **(G)** PET indicated low metabolism in the right cerebral hemisphere. **(H)** The PFV of RACA, RMCA, and RPCA was 107, 71, and 78 cm/s, respectively. **(I)** The PFV of LACA was 110 cm/s, LMCA was 127 cm/s, and LPCA was 64 cm/s. **(B,C)** were case **(A)**; **(E,F)** were case **(D)**; **(H,I)** were case **(G)**; R, right; L, left; ACA, anterior cerebral artery; MCA, middle cerebral artery; PCA, posterior cerebral artery.

Patients were divided into three groups according to the arterial blood supply corresponding to the low metabolism range of PET. The first group was a single arterial supply group. This group consisted of nine patients with low metabolism in the blood supply area of the unilateral MCA (Table 2, Figure 2A). The average PFV and MFV of the abnormal side MCA were 88 and 56 cm/s, respectively, and the average PFV and MFV of the corresponding side MCA were 88 and 56 cm/s, respectively (Tables 2, 3, Figures 2B,C). One patient had low metabolism in the blood supply area of unilateral PCA, the average PFV and MFV of the abnormal side PCA were 62 and 35 cm/s, respectively, and the average PFV of the corresponding side PCA were 69 and 44 cm/s, respectively (Tables 2, 3). In the second group, there were three patients with low metabolism in the blood supply areas of the MCA and the PCA (Table 2, Figure 2D). The average PFV of the abnormal side MCA and PCA was 98 and 54 cm/s, respectively; the average PFV of their corresponding MCA and PCA was 101 and 70 cm/s, respectively. In addition, The average MFV of the abnormal side MCA and PCA was 59 and 34 cm/s, respectively; the average MFV of their corresponding MCA and PCA was 63 and 45 cm/s, respectively (Tables 2, 3). The PFV and MFV of the PCA on the abnormal side were lower than that on the opposite side, but these differences did not achieve statistical significance (Tables 2, 3, Figures 2E,F). In the third group, there were two patients with low metabolism in the blood supply regions of the ACA, MCA, and PCA (Table 2, Figure 2G). In one patient with extensive hypometabolism in the right hemisphere, the PFV and MFV of the ipsilateral MCA with hypometabolism were lower than those of the contralateral MCA (Tables 2, 3, Figures 2H,I).

**Table 3:** The peak flow velocity (PFV) and mean flow velocity (MFV) of the cases.

**Case number**	**PFV (cm/s)**						**MFV (cm/s)**					
	**L-ACA**	**L-MCA**	**L-PCA**	**R-ACA**	**R-MCA**	**R-PCA**	**L-ACA**	**L-MCA**	**L-PCA**	**R-ACA**	**R-MCA**	**R-PCA**
1	83	97	47	95	93	48	52	60	31	63	56	30
2	81	95	39	58	85	46	48	56	25	39	54	28
3	87	90	64	92	111	51	51	56	39	58	63	32
4	90	104	67	76	86	37	61	65	44	52	52	24
5	69	83	67	71	78	59	46	55	39	48	49	41
6	110	127	64	107	71	78	71	79	43	57	48	27
7	62	75	62	74	73	69	38	48	35	43	43	44
8	78	118	50	108	118	58	23	72	29	66	70	36
9	96	98	53	97	96	64	66	64	34	65	64	44
10	78	76	42	71	80	44	51	51	27	45	55	29
11	102	114	63	92	97	73	62	67	38	58	62	48
12	62	64	42	33	67	36	40	44	42	22	44	23
13	63	70	48	65	76	54	38	45	32	32	49	37
14	46	109	47	69	98	49	26	67	29	44	58	31
15	60	101	71	55	85	48	36	61	46	30	55	29
16	81	103	69	68	93	62	50	62	43	40	58	39

*L, left; R, right; ACA, anterior cerebral artery; MCA, middle cerebral artery; PCA, posterior cerebral artery*.

**Figure 3:**
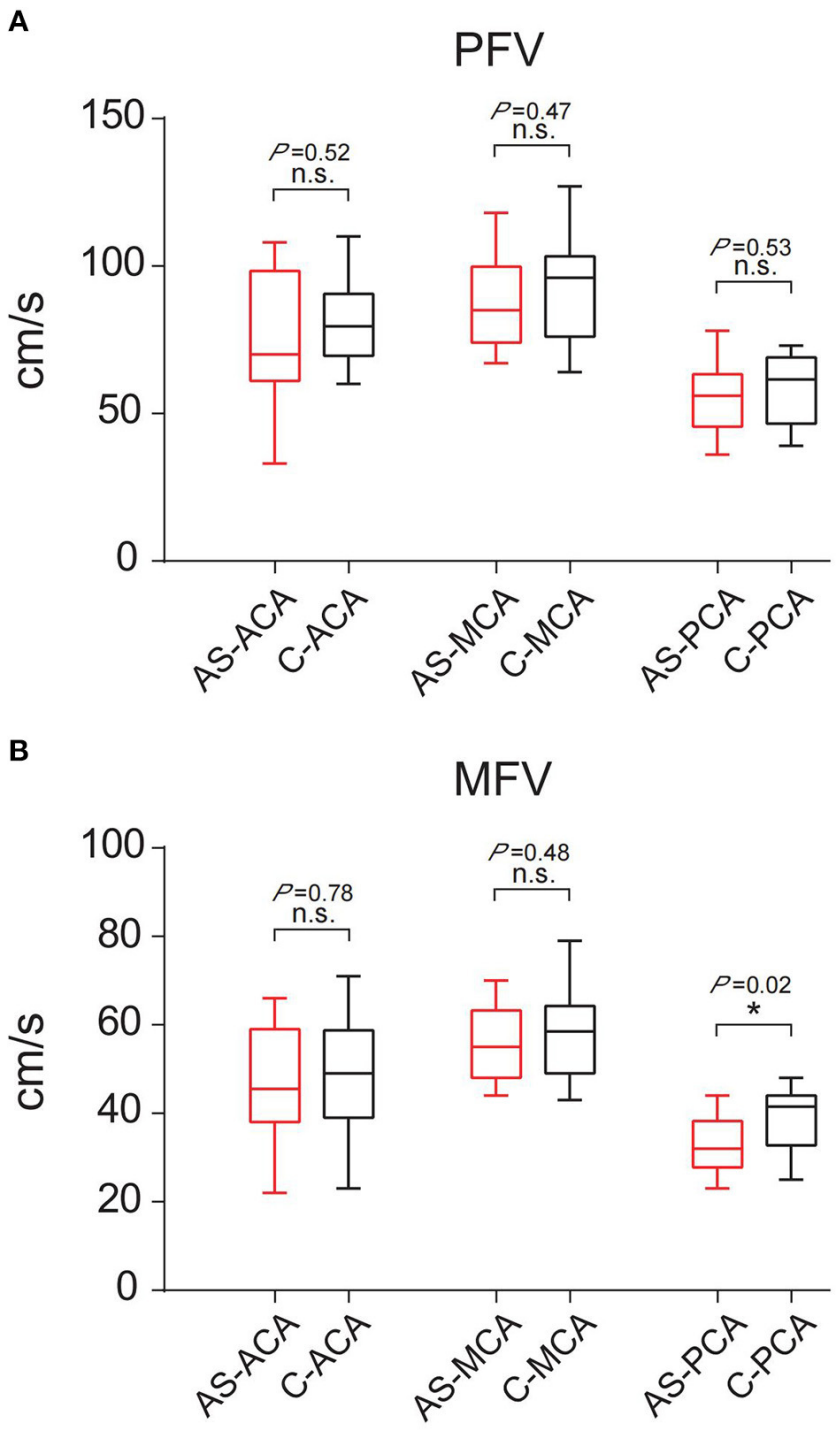
Comparison of PFV and MFV of the corresponding vessels on the low metabolic side and contralateral side of PET. **(A)** PFV. **(B)** MFV. “*”: *P* < *0.05*; n.s., no statistical significance; AS, abnormal side; C, contralateral; ACA, anterior cerebral artery; MCA, middle cerebral artery; PCA, posterior cerebral artery.

A comparison of the flow velocity (PFV and MFV) on the low metabolic side and the contralateral side of the PET showed the average PFV of the ACA, MCA, and PCA on the abnormal side was 76, 88, and 55 cm/s, respectively; the average MFV was 47, 56, and 33 cm/s, respectively. The average PFV of the ACA, MCA, and PCA on the corresponding side was 80, 93, and 58 cm/s, respectively; the average MFV was 48, 58, and 39 cm/s, respectively. The PFV and MFV's of each of the three arteries on the abnormal side were lower than that on the contralateral side. The difference in PCA's MFV was statistically significant (*P* < *0.05*) (Figure 3).

### Interictal EEG Patterns and Flow Velocity of the Corresponding Bilateral Arteries

**Figure 4:**
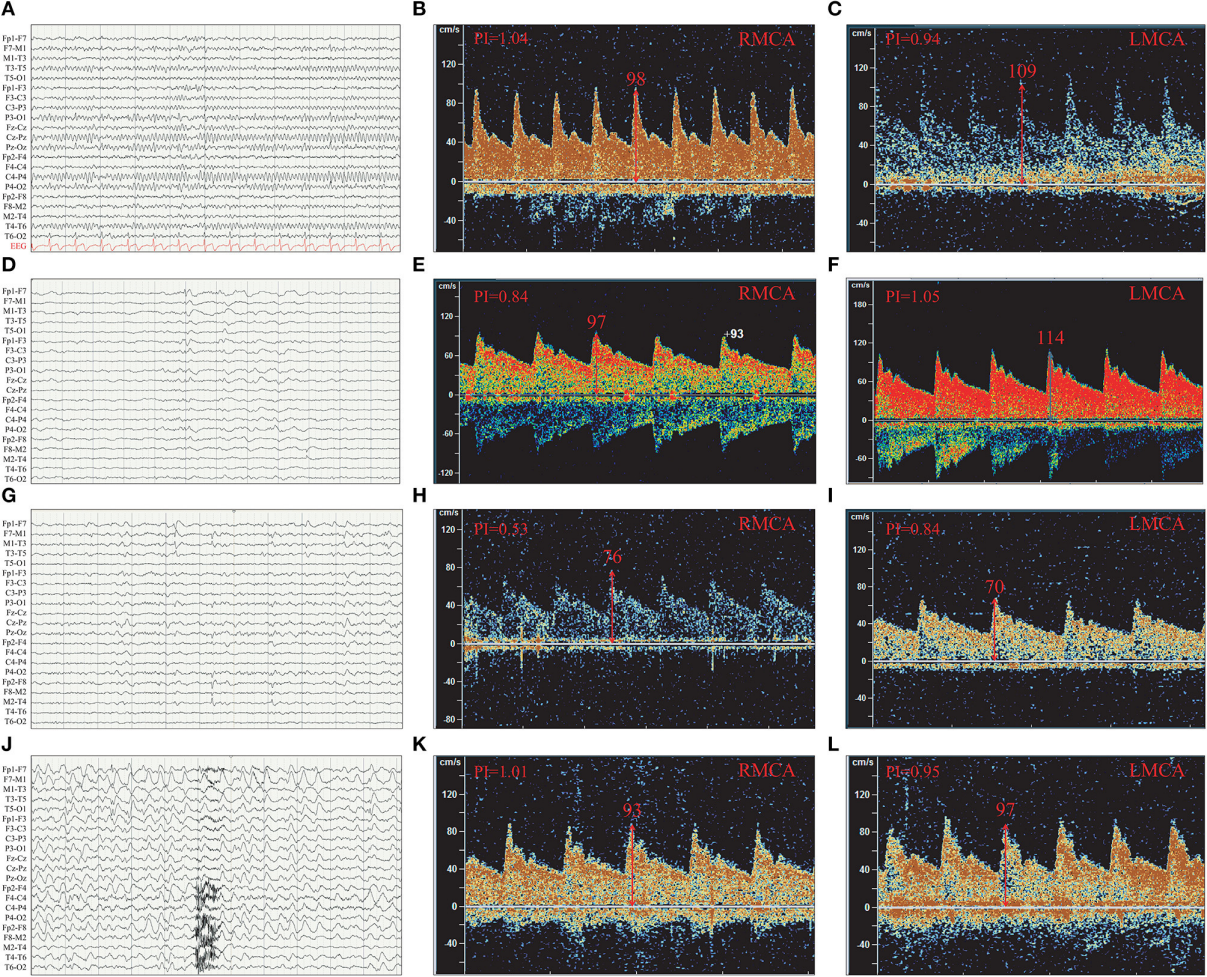
The PFV of the left and right MCA were compared according to interictal EEG discharge patterns. **(A)** Normal EEG. **(B)** The PFV of RMCA was 98 cm/s. **(C)** The PFV of LMCA was 109 cm/s. **(D)** Left temporal discharge (unilateral discharge pattern); **(E)** The PFV of RMCA was 97 cm/s. **(F)** The PFV of LMCA was 114 cm/s. **(G)** Bilateral temporal asynchronous discharge (bilateral discharge pattern); **(H)** The PFV of RMCA was 76 cm/s. **(I)** The PFV of LMCA was 70 cm/s. **(J)** Bilateral multi-brain areas discharge (bilateral discharge pattern); **(K)** The PFV of RMCA was 93 cm/s. **(L)** The PFV of LMCA was 97 cm/s. **(B,C)** were case **(A)**; **(E,F)** were case **(D)**; **(H,I)** were case **(G)**; **(K,L)** were case **(J)**; R, right; L, left; ACA, anterior cerebral artery; MCA, middle cerebral artery; PCA, posterior cerebral artery.

**Figure 5:**
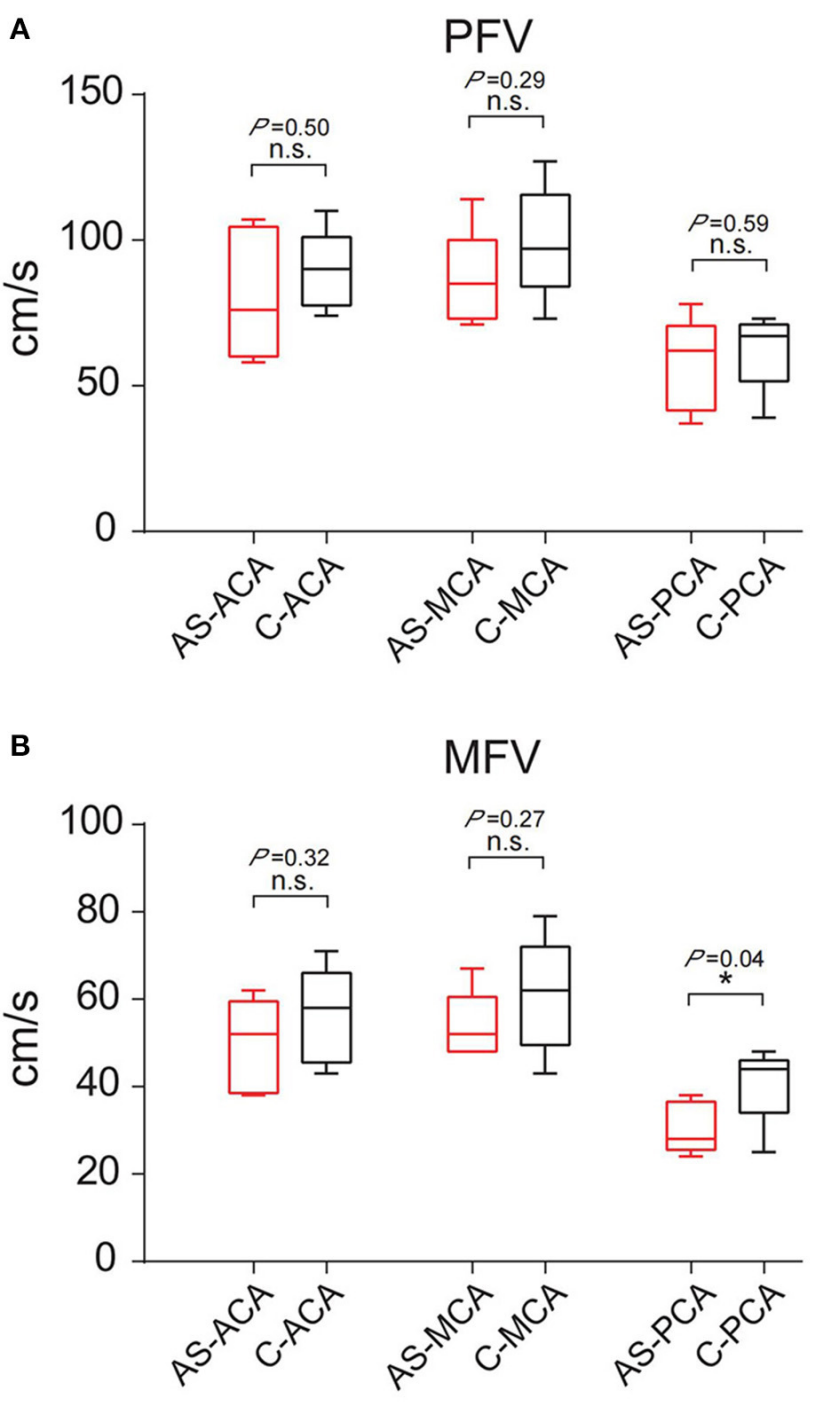
Comparison of PFV and MFV of the corresponding vessels on the interictal discharge side and contralateral side. **(A)** PFV. **(B)** MFV. “*”: *P* < *0.05*; n.s., no statistical significance; AS, abnormal side; C, contralateral; ACA, anterior cerebral artery; MCA, middle cerebral artery; PCA, posterior cerebral artery.

The 16 patients were divided into three groups according to the discharge patterns of interictal EEG. One patient's EEG showed no definite abnormal discharge (Tables 2, 3, Figures 4A–C). There were five patients with unilateral discharge (Table 2, Figure 4D). Their average PFV of the ACA, MCA, and PCA on the abnormal side was 81, 86, and 57 cm/s, respectively; the average PFV of the ACA, MCA, and PCA on the corresponding side was 89, 99, and 62 cm/s, respectively. Their average MFV of the ACA, MCA, and PCA on the abnormal side was 50, 54, and 30 cm/s, respectively; the average MFV of the ACA, MCA, and PCA on the corresponding side was 56, 61, and 41 cm/s, respectively (Tables 2, 3, Figures 4E,F). The PFV and MFV's of each of the three arteries on the abnormal side were lower than that on the contralateral side. The difference in PCA's MFV was statistically significant (*P* < *0.05*) (Figure 5). There were 10 patients who had a bilateral discharge on their EEG (Table 2, Figures 4G,J). Their average PFV of the left ACA, MCA, and PCA was 76, 90, and 55 cm/s, respectively; the average PFV of the right ACA, MCA, and PCA was 76, 90, and 52 cm/s, respectively. Their average MFV of the left ACA, MCA, and PCA was 44, 58, and 36 cm/s, respectively; the average MFV of the right ACA, MCA, and PCA was 47, 56, and 34 cm/s, respectively (Tables 2, 3, Figures 4H,I,K,L). There was no significant difference in the PFV or MFV of the corresponding vessels on either side.

**Figure 6:**
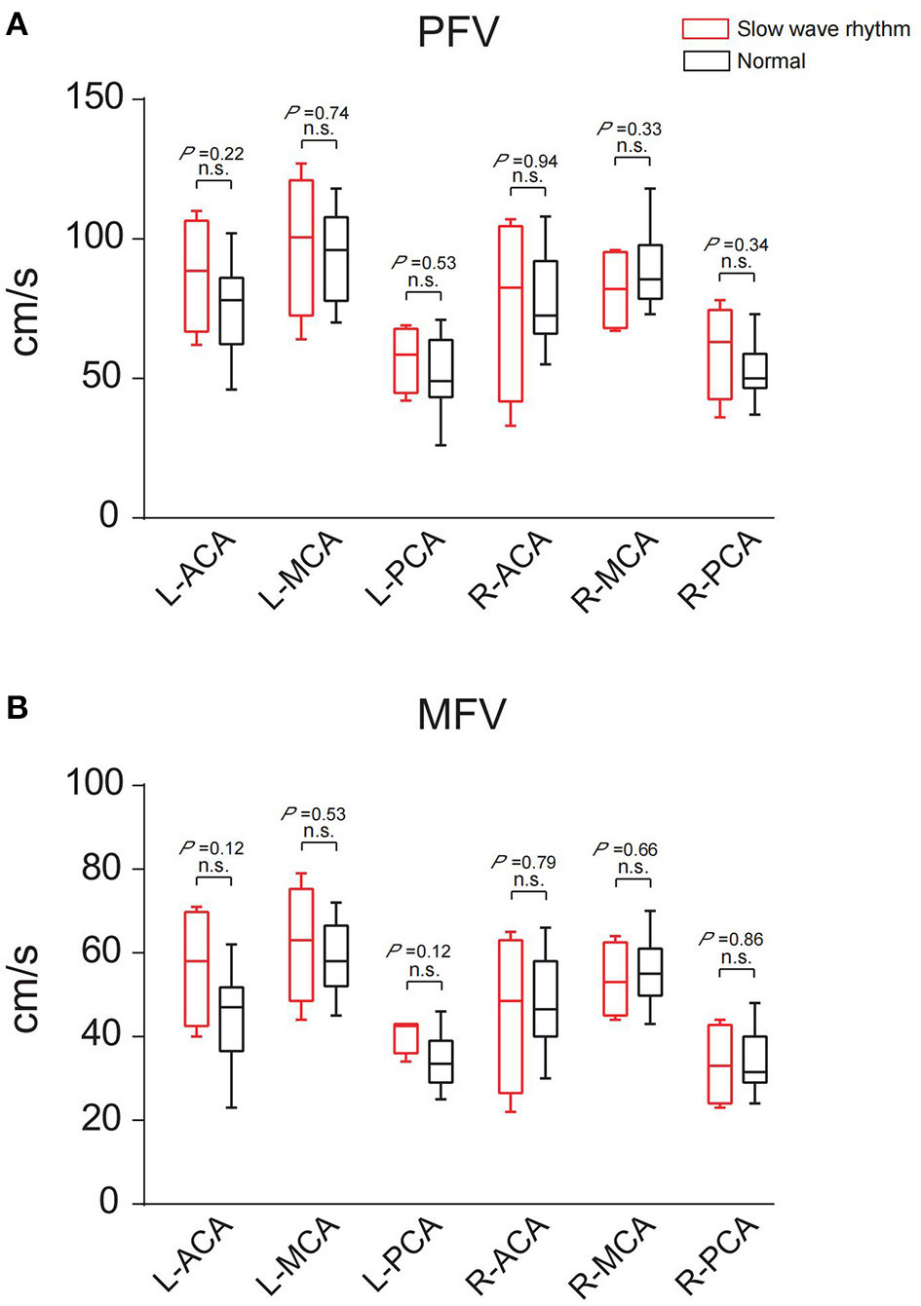
The PFV and MFV of the same blood vessels in the two groups with slow background rhythm and normal background rhythm were compared. **(A)** PFV. **(B)** MFV. n.s., no statistical significance; R, right; L, left; ACA, anterior cerebral artery; MCA, middle cerebral artery; PCA, posterior cerebral artery.

Among the 16 patients, four had a decreased background rhythm (Table 2). Their average PFV of the left ACA, MCA, and PCA was 87, 98, and 57 cm/s, respectively; the average PFV of the right ACA, MCA, and PCA was 76, 82, and 60 cm/s, respectively. Their average MFV of the left ACA, MCA, and PCA was 57, 62, and 41 cm/s, respectively; the average MFV of the right ACA, MCA, and PCA was 46, 54, and 33 cm/s, respectively (Tables 2, 3). The other 12 patients had a normal background rhythm (Table 2). The average PFV of their left ACA, MCA, and PCA was 75, 94, and 52 cm/s, respectively; the average PFV of the right ACA, MCA, and PCA was 77, 90, and 53 cm/s, respectively. Their average MFV of the left ACA, MCA, and PCA was 44, 59, and 35 cm/s, respectively; the average MFV of the right ACA, MCA, and PCA was 48, 56, and 34 cm/s, respectively (Tables 2, 3). There was no obvious rule between the PFV or MFV of the corresponding vessels in the background slowed rhythm group and the normal group. Additionally, the difference between the values was not statistically significant (Figure 6).

These results suggested that PFV and MFV of the main cerebral vesselson the low metabolic side of PET or the interictal discharge side of EEG were decreased compared with the contralateral side, especially in the PCA.

## Discussion

In this study, the PFV and MFV of the main vessels in the brain of patients with epilepsy were measured and compared between the left and right sides. On the whole, the PFV and MFV of the ACA, MCA, and PCA of the brain with low metabolic abnormalities were lower than that of the corresponding sides. There was no statistically significant difference in the PFV of the corresponding blood vessels on both sides for neither the blood supply vessels corresponding to the low metabolism range of PET nor between the low metabolism side and the contralateral side. Low metabolism of PET is a commonly used imaging marker of refractory epilepsy, which reflects decreased glucose metabolism uptake (5). The mechanism is still unclear for epilepsy. Previous studies have shown that the low metabolic manifestations of epileptogenic regions in epilepsy patients may be related to the decline of mitochondrial IV function (6) and may also be related to the abnormal energy metabolism of glial cells (7). Previous studies using PET during seizures (8–10) have shown that PET demonstrated high metabolism in epileptic regions during seizures, suggesting that the ability of glucose utilization in epileptic regions still exists.

In addition, we also analyzed the differences in PFV and MFV under different interictal discharge patterns in this study. We observed no statistically meaningful difference in the PFV between the interictal discharge side and the opposite side or whether the EEG background slowed down. Since none of the patients had vascular stenosis there was no significant decrease in blood supply to the epileptogenic zone, suggesting that patients with epilepsy may have a selective decrease in glucose utilization in the epileptogenic zone during the interictal period. It is well-known that epilepsy occurs and develops as a network of neurons that can affect a large area of the brain. During the interictal period, neuronal excitability is high, and each seizure can be understood as a transmission process of high-energy electrical activity along specific neural fiber pathways which belong to long-range neural network projection (11). This means that a higher energy supply (ATP) is required to maintain the high neuronal excitability levels during both the interictal and the ictal phases. Previous studies on ketogenic diets have shown that brain tissue using ketones not only provides more energy but also reduces the excitability of neurons, leading to seizure control (12–14). Gimenez-Cassina et al. (15) confirmed that in the absence of any dietary influence, the selective conversion of brain energy consumption from glucose to ketone body is associated with BAD dependent metabolic transfer and affect the excitability of neurons in patients with epilepsy. Combined with the results of this study and previous studies, in epilepsy patients with constant or reduced cerebral hemodynamics, ketone body, rather than glucose, may be selectively utilized by brain tissues in the epileptogenic zone to increase energy supply and reduce neuronal excitability. This was followed by low metabolism on PET examination.

A limitation of this study is its small sample size; this likely accounts for the lack of statistical significance in the PFV and MFV differences. Although the PFV in the low metabolism area of PET and the interictal discharge area of EEG decreased compared with the contralateral side, the difference was not statistically significant. However, the MFV of PCA on the low metabolic side of PET or the interictal discharge side was significantly different from that of the contralateral vessels. Most of the patients enrolled in this study were hypometabolism and discharge in the temporal region, and PCA was an important blood vessel supplying the hippocampus and the medial temporal lobe (16), and the hemodynamic changes in this region were consistent with the epileptogenic zone. Our results suggest that MFV is a better parameter than PFV in hemodynamic assessment of epilepsy, and the evaluation of cerebral hemodynamics may be helpful for the lateral and localized diagnosis of epilepsy. Future studies with a larger cohort and perhaps even multi-center studies are needed to further study this process and its molecular mechanism.

## Conclusion

In this study, we found that the PFV and MFV values of the PET hypometabolized side and the interictal EEG discharge side of epileptic patients were lower than the values of the corresponding blood vessels on the contralateral side. To some extent, the difference in the MFV of PCA between the bilateral sides can assist the lateral diagnosis of the epileptogenic zone.

## Data Availability Statement

The original contributions presented in the study are included in the article/supplementary material, further inquiries can be directed to the corresponding author/s.

## Ethics Statement

The studies involving human participants were reviewed and approved by The Ethics Committee of Shengjing Hospital of China Medical University. Written informed consent to participate in this study was provided by the participants' legal guardian/next of kin. Written informed consent was obtained from the individual(s), and minor(s)' legal guardian/next of kin, for the publication of any potentially identifiable images or data included in this article.

## Author Contributions

All authors were involved in the study design, interpretation of the results, and the reviewing and approval of the manuscript, and in the decision to submit the article for publication. All authors also confirm accountability for the accuracy and integrity of the work.

## Conflict of Interest

The authors declare that the research was conducted in the absence of any commercial or financial relationships that could be construed as a potential conflict of interest.
